# Easy-to-use and easy-to-interpret quality control of 3D gradient echo T1-weighted MR acquisition sequences for improved test-retest stability of MRI-based hippocampus volumetry

**DOI:** 10.1177/13872877251380301

**Published:** 2025-09-24

**Authors:** Ralph Buchert, Per Suppa, Babak A Ardekani, Fuensanta Bellvís Bataller, Pierrick Bourgeat, Pierrick Coupé, Robert Dahnke, Gabriel A Devenyi, Simon Fristed Eskildsen, Clara Fischer, Jose Vincente Manjón Herrera, Christian Ledig, Andreas Lemke, Bénédicte Maréchal, Roland Opfer, Diana M Sima, Lothar Spies, Aziz M Ulug, Hans-Jürgen Huppertz

**Affiliations:** 1 Department of Diagnostic and Interventional Radiology and Nuclear Medicine, University Medical Center Hamburg-Eppendorf, Hamburg, Germany; 2 Olympus Winter & Ibe GmbH, Hamburg, Germany; 3 Center for Brain Imaging and Neuromodulation, The Nathan S. Kline Institute for Psychiatric Research, Orangeburg, NY, USA; 4 Quantitative Imaging Biomarkers in Medicine (Quibim), Valencia, Spain; 5 Australian e-Health Research Centre, CSIRO Health and Biosecurity, Brisbane, Australia; 6 LaBRI – UMR 5800, University of Bordeaux, Talence, France; 7 Structural Brain Mapping Group, Department of Neurology, Jena University Hospital, Jena, Germany; 8 Department of Psychiatry and Psychotherapy, Jena University Hospital, Jena, Germany; 9 German Center for Mental Health (DZPG), Jena, Germany; 10 Department of Psychiatry, Cerebral Imaging Center, Douglas Mental Health University Institute, McGill University, Montréal, Quebec, Canada; 11 Department of Clinical Medicine, Center of Functionally Integrative Neuroscience, Aarhus University, Aarhus, Denmark; 12 CATI, US52-UAR2031, CEA, ICM, SU, CNRS, INSERM, APHP, Ile de France, Paris, France; 13 Applied Physics Department, MIA Lab, ITACA Institute, Universidad Politécnica de Valencia, Valencia, Spain; 14 xAILab Bamberg, University of Bamberg, Bamberg, Germany; 15 Mediaire GmbH, Berlin, Germany; 16 Advanced Clinical Imaging Technology, Siemens Healthineers International AG, Lausanne, Switzerland; 17 Jung Diagnostics GmbH, Hamburg, Germany; 18 Icometrix, Leuven, Belgium; 19 Cortechs Labs, Inc., San Diego, CA, USA; 20 Swiss Epilepsy Center, Klinik Lengg, Zurich, Switzerland

**Keywords:** Alzheimer's disease, classification-and-regression tree, contrast-to-noise ratio, field-of-view, hippocampus, image quality, outlier, structural MRI, volumetry

## Abstract

**Background:**

MRI-based hippocampus volume (HV) is widely used as neurodegeneration marker in Alzheimer's disease.

**Objective:**

An easy-to-use and easy-to-interpret method to categorize T1-weighted MR sequences with respect to test-retest stability of hippocampus volumetry based on general image quality metrics (IQM).

**Methods:**

The study included 446 3D T1-weighted MRI scans of one healthy middle-aged man obtained during 32 months in 122 scanning sessions performed with 96 different scanners at 76 different sites. Each scanning session represented a different acquisition sequence of ≥2 back-to-back repeat scans (3.7 ± 0.7 on average). Unilateral HVs were determined with 18 different tools for automatic volumetry. An acquisition sequence was considered “poor” if the z-score of the within-session coefficient-of-variation of the HV estimates from the session, averaged across all volumetry tools and both hemispheres, exceeded one standard deviation. General IQM were computed for each scanning session using the freely available MRI Quality Control Tool. A classification-and-regression tree (CART) was trained to discriminate between good and poor acquisition sequences using the IQM as input.

**Results:**

The CART selected the left-right width of the acquisition field-of-view and the contrast-to-noise ratio as predictor variables. Overall accuracy of the CART was 79.5%. CART-based classification increased the ratio of good-to-poor acquisition sequences from 3.5 among all sequences to 7.4 among the sequences predicted to be good. This was at the expense of losing 15% of the good sequences.

**Conclusions:**

The IQM-based decision tree model provides useful performance for the differentiation of T1-weighted sequences associated with good versus poor test-retest stability of hippocampus volumetry.

## Introduction

Detection (or exclusion) of global and regional atrophy using MRI-based brain volumetric estimates is increasingly used in everyday clinical routine to assist the diagnosis, differentiation and monitoring of neurodegenerative diseases.^1–4^ In the diagnostic workflow of Alzheimer's disease (AD), hippocampus volume (HV) estimated from high-resolution 3-dimensional (3D) gradient-echo T1-weighted structural MRI (sMRI) is used as a supportive diagnostic biomarker,^5–10^ to stage neuronal damage,^11,12^ and to predict progression to dementia from prodromal disease stages.^13–15^

However, MRI-based volumetry is sensitive to the setting (scanner platform & acquisition sequence), resulting in non-biological variability of no interest.^16–21^ In particular, the utility of MRI-based brain volumetry can be affected by low image quality. Relevant degradation of image quality in *individual* scans can be caused by subject-related factors including motion during the acquisition.^22–24^ Systematically low image quality of *all* scans from a given source can be associated with hardware-specific factors (that cannot be changed) and with details of the acquisition sequence (that can be adjusted). Low image quality can be reflected by poor signal-to-noise ratios,^25^ intensity non-uniformity, spatial image distortion, and substantial deviation from the standard white-to-gray matter contrast.^17^

In large scale imaging research collecting MRI scans across numerous sites,^26–28^ the statistical power may be reduced by large test-retest variability of some “poor” acquisition sequences with low image quality. In clinical practice, large variability of HV estimates can lead to erroneous conclusions^29^ that may negatively impact patient management. Thus, identification and exclusion of acquisition sequences associated with large test-retest variability of HV estimates may have considerable impact in both research and clinical practice.

Low image quality associated with hardware- and sequence-specific factors can be detected from scans of physical/geometrical phantoms.^30–32^ However, these phantoms may not allow reliable prediction of test-retest stability with respect to very specific tasks such as hippocampus volumetry. Scanning healthy subjects, serving as human phantoms, can be more appropriate.^18,33–40^ General image quality metrics (IQM) derived from normal human scans have been used for retrospective harmonization of volumetric MRI data across scanners and acquisition sequences.^41^ It is plausible, therefore, to assume that general IQM are useful also for the characterization of acquisition sequences with respect to test-retest stability of hippocampus volumetry.

Against this background, the aim of the current study was to develop an easy-to-use and easy-to-interpret method that uses general IQM derived from human phantom scans to distinguish between sMRI acquisition sequences with high versus low test-retest stability of hippocampus volumetry.

There was a strong focus on generalizability to data from unseen scanners and unseen volumetry tools. To address this, the study used 18 different volumetry tools, including commercial and research tools, and a subset of the frequently traveling human phantom (FTHP) dataset. The latter provides 557 sMRI scans of a single healthy male volunteer who completed 176 scanning sessions on 116 different MRI scanners at 96 different sites.^42^ In each scanning session, all back-to-back repeat scans were acquired with exactly the same sequence parameters. Thus, each scanning session represents a different acquisition setting (MRI scanner & acquisition sequence). The MRI Quality Control Tool (MRIQC),^29^ which is freely available both for local implementation and as an online service, was used to compute general IQM as input to the prediction model.

## Methods

### Frequently traveling human phantom (FTHP) dataset

The FTHP dataset has been recently introduced and made freely available by our group^42^ (https://www.kaggle.com/datasets/ukeppendorf/frequently-traveling-human-phantom-fthp-dataset). It provides 557 sMRI scans of a single healthy male (48.6 years old at the first scan) who completed 176 scanning sessions on 116 different MRI scanners at 96 different sites. Most of the sites were private radiological practices and small hospitals. The scanning sessions were performed during a period of 32 months (07/2017–02/2020). In each scanning session, 1–6 (median 4) sMRI were acquired consecutively without repositioning and without delay using the same acquisition sequence. Different scanning sessions from the same MRI scanner differed with respect to the acquisition sequence. Thus, each scanning session represents a different acquisition setting, that is, a different combination of MRI scanner and acquisition sequence. In the following, we will use “acquisition sequence” to denote “combination of MRI scanner and acquisition sequence” to simplify the notation.

All imaging sites were informed that the scans were being acquired for the purpose of MRI-based volumetry. They were asked to use acquisition sequences recommended by the scanner manufacturer. Thus, the FTHP dataset can be considered representative of MRI-based volumetry in everyday clinical routine at non-academic sites. At the time of writing this manuscript (02/2025), more than 4 years after the last scan, the volunteer is still in excellent health.

For the analyses reported in this manuscript, 26 of the 557 sMRI scans were excluded because they were secondary images from the same acquisition (derived from the primary images by post-processing), 3 scans were excluded because of inconsistent DICOM header information (slice location attribute), and one scan was excluded because it was not acquired with a 3D sequence (Supplemental “Excluded scans”). The remaining 527 sMRI scans were submitted to automatic hippocampus volumetry.

The subject provided written informed consent for the retrospective use of the FTHP dataset for research purposes. This was approved by the ethics review board of the general medical council of the state of Hamburg, Germany (reference number PV5930).

The cost of travel and MR scanning for the FTHP dataset was covered by a private company, specializing in quantitative analyses of medical images (MRI, SPECT and PET) for use in clinical practice and drug trials, in order to qualify the structural MRI acquisition sequences used by their customers for MRI-based brain volumetric analyses. As the FTHP dataset was used retrospectively in the current study, no funding was required.

### Automatic hippocampus volumetry

The HV in left and right hemispheres were determined for each scan independently with 19 different tools for automatic MRI-based volumetry, including both commercial and research tools (Table 1). The MRI-based volumetry was performed non-centrally by the providers/developers of the volumetry tools. For this purpose, the sMRI scans were pseudonymized in fully randomized order (i.e., without grouping of scans from the same session) and made available to the providers/developers of the volumetry tools in both DICOM and NIFTI format. Each provider/developer returned the HV estimates as a spreadsheet table for central analysis.

**Table 1.** Tools used for automatic hippocampus volumetry. The tools are listed in random order not matching the alphabetical order of the pseudonyms A, …, V.

Tool	Method	Publications	Website
mdbrain AI	convolutional neural network (deep learning)	^43–45^	www.mediaire.de/en/home
AI-Rad Companion Brain MR version VA40	single-atlas	^46,47^	www.siemens-healthineers.com/en-sg/digital-health-solutions/digital-solutions-overview/clinical-decision-support/ai-rad-companion/brain-mr
MINC Toolkit 2	multi-atlas (patch-based)	^48^	github.com/BIC-MNI/minc-toolkit-v2
KAIBA	automatic landmark detection and histogram analysis on the VOI	^49–51^	www.nitrc.org/projects/art
ABV (Atlas-based Volumetry)	single-atlas	^19,52,53^	https://kliniklengg.ch/schweizerisches-epilepsie-zentrum/diagnostik/medizinische-bildverarbeitung-mri/volumetrische-mri-analyse
CurAIBL	multi-atlas	^54,55^	milxcloud.csiro.au/tools/curaibl
mdbrain atlas	atlas-based	^56^	www.mediaire.de/en/home
icobrain dm 5.4	multi-atlas	^57^	www.icometrix.com
Quibim Precision	multi-atlas	^58,59^	www.quibim.com
SACHA	multi-template	^60–62^	
CAT12	multi-atlas		neuro-jena.github.io/cat/
volbrain	multi-atlas	^63^	volbrain.net/
Neuroreader	multi-atlas	^64^	brainreader.net
icobrain dl 5.15	convolutional neural network (deep learning)	^65^	www.icometrix.com
MALPEM	multi-atlas	^66–68^	github.com/ledigchr/MALPEM
Biometrica 1.1	single-atlas	^8,15^	www.jung-diagnostics.de/
Freesurfer 7	single-atlas	^69,70^	surfer.nmr.mgh.harvard.edu/
NeuroQuant	dynamic atlas	^71,72^	www.cortechs.ai/
MAGeT-Brain	modified multi-atlas	^73^	github.com/CoBrALab/MAGeTbrain

All volumetry tools provided HV in ml (except one tool that provided the hippocampal parenchymal fraction). The raw volumes in ml were used throughout this study without any correction or scaling (e.g., with respect to the total intracranial volume, age or sex).

### Within-session test-retest variability of HV estimates

First, the following two steps were performed separately for each volumetry tool and each hemisphere:

- For each scanning session, the within-session coefficient of variation (CoV = 100 * standard deviation / mean) of the HV estimates was computed across the 2 to 6 back-to-back scans in this session.
- The within-session CoVs were transformed to z-scores relative to the mean and standard deviation of the within-session CoV across the different scanning sessions. Outlier sessions with respect to the within-session CoV were excluded from the computation of mean and standard deviation. A scanning session was considered an outlier if its within-session CoV was greater than CoV_3_ + 1.5 × IQR or less than CoV_1_ − 1.5 × IQR, where CoV_1_ and CoV_3_ denote the first and third quartiles of the within-session CoV across all scanning sessions and IQR (interquartile range) is CoV_3_-CoV_1_.^74^ The Box-Cox transformation determined on the non-outlier sessions was applied prior to the z-score transformation to reduce non-normality effects.

Second, the z-scores were averaged across all volumetry tools and both hemispheres, separately for each scanning session. The resulting mean within-session CoV z-score of a given scanning session characterizes the acquisition sequence used in this scanning session with respect to the within-session test-retest variability of HV estimates independent of a specific volumetry tool.

### Poor scanning sessions/acquisition sequences

The distribution of the mean within-session CoV z-score across the scanning sessions was fitted by a Gaussian function. The mean value of the Gaussian was fixed at z-score = 0. The standard deviation (SD) of the resulting Gaussian was used to define a cutoff on the mean within-session CoV z-score to discriminate between “poor” and “good” scanning sessions. More precisely, a scanning session was considered “poor” if its mean within-session CoV z-score was larger than +1.0*SD (of the Gaussian), otherwise it was considered “good” (mean within-session CoV z-score ≤ + 1.0*SD). An *acquisition sequence* was considered “poor” or “good” according to the categorization of the corresponding scanning session. “Poor” acquisition sequences result in worse (within-session) stability of HV estimates compared with “good” acquisition sequences.

### General image quality control metrics

The MRI Quality Control Tool (MRIQC, version 22.0.6)^29,75^ was used to compute the contrast-to-noise ratio (CNR) and the full-width-at-half-maximum according to the Analysis of Functional NeuroImages software package^76^ (AFNI-FWHM) for each sMRI scan (average of 3dFWHMx, 3dFWHMy, 3dFWHMz). The CNR characterizes the separation of gray matter and white matter tissue signals relative to the noise level (larger values indicate better quality).^77^ It is computed as $CNR=|{\hat{\mu }}_{GM}-{\hat{\mu }}_{WM}|/$ $\sqrt{{\hat{\sigma }}_{GM}^{2}+{\hat{\sigma }}_{WM}^{2}+{\hat{\sigma }}_{Air}^{2}}$ , where $\hat{\mu }$ and ${\hat{\sigma }}^{2}$ indicate the estimated mean value and variance of the signal intensity in the gray matter (GM), white matter (WM), and in the air background surrounding the head (mriqc.readthedocs.io/en/latest/iqms/t1w.html). The CNR has been used previously to assess the association between image quality and brain volumetric measurements.^33,56^ The AFNI-FWHM characterizes the noise smoothness of the images (smaller values indicate better quality).

The within-session means of CNR and AFNI-FWHM across all back-to-back repeat scans in a given scanning session were used to characterize the image quality of the corresponding acquisition sequence.

In addition, the width of the acquisition field-of-view (FOV) in left-right direction (FOVx) and in anterior-posterior direction (FOVy) were used to characterize acquisition sequences. The rationale for this was that a narrow field-of-view just barely including the whole head might be associated with edge artefacts that negatively impact automatic volumetry.

### Statistical analysis

The aim was to discriminate between good and poor acquisition sequences based on the session means of CNR and AFNI-FWHM and on FOVx and FOVy.

Decision tree analysis using a classification and regression tree (CART) was utilized for this purpose. The growth method of this decision tree variant aims at minimum intra-node impurity, that is, maximum intra-node homogeneity (optimally, all cases of a terminal node belong to the same category). The measure of impurity to be minimized by the CART was the Gini-Simpson diversity index, which is the standard for categorical dependent variables. The Gini-Simpson index of a node characterizes the rate of false categorization when randomly selected cases are randomly categorized according to the distribution of the categories within the node.^78^ The minimum improvement in the Gini-Simpson index required to split a node was set to 0.02. The minimum size of parent and child nodes at each tree branching was set to 20 and 3, respectively. The maximum depth of the CART was set to 2. The CART was free to select the predictor for the first split (CNR, AFNI-FWHM, FOVx or FOVy), that is, it was not forced to select a specific predefined predictor first. The CART was trained with 5-fold cross-validation.

The impact of the CART-based categorization of acquisition sequences on the within-session stability of hippocampus volume estimates was tested by repeated measures analysis of variance (ANOVA) of the within-session CoV using volumetry tool (A, …, V) and hemisphere (left, right) as within-session factors and the CART-based categorization (good session, poor session) as between-session factor.

## Results

Twenty-two (4.2%) of the 527 sMRI scans were excluded from the analyses due to non-acceptable image quality according to visual inspection (Supplemental “Excluded scans”): 5 (0.9%) scans were excluded due to very low gray-to-white matter contrast (Supplemental Figure 1a), 12 (2.3%) due to a severe bias field (Supplemental Figure 1b), and 5 (0.9%) sMRI scans were excluded due to a narrow field-of-view not including the entire head (Supplemental Figure 1c). From the remaining 505 sMRI scans, 35 (6.9%) were excluded because HV estimates were not provided for all of the 19 volumetry tools (HV estimates missing for 4/22/4/2/8 scans for tool C/O/P/Q/U). From the remaining 470 sMRI scans, 24 (5.1%) single scans were excluded (no back-to-back repeat scans in the same scanning session). The remaining 446 sMRI scans were included in the analyses. These had been acquired in 122 different scanning sessions with 96 different MRI scanners at 76 different sites (Supplemental Table 1). The number of back-to-back scans was 2 in 8 (6.6%) of the 122 scanning sessions, 3 in 37 (30.3%) sessions, 4 in 67 (54.9%) sessions, 5 in 9 (7.4%) sessions, and 6 in one (0.8%) scanning session. The MRI scanner manufacturer was Siemens in 74 scanning sessions (60.7%), Philips in 36 sessions (29.5%), and GE in 12 sessions (9.8%). The magnetic field strength (B_0_) was 1.5 Tesla in 87 scanning sessions (71.3%), 3 Tesla in 34 sessions (27.9%), and 1 Tesla in one session (0.8%). Slice orientation was sagittal in 83 scanning sessions (68.0%), axial in 35 sessions (28.7%), and coronal in 4 sessions (3.3%).

**Figure 1.**
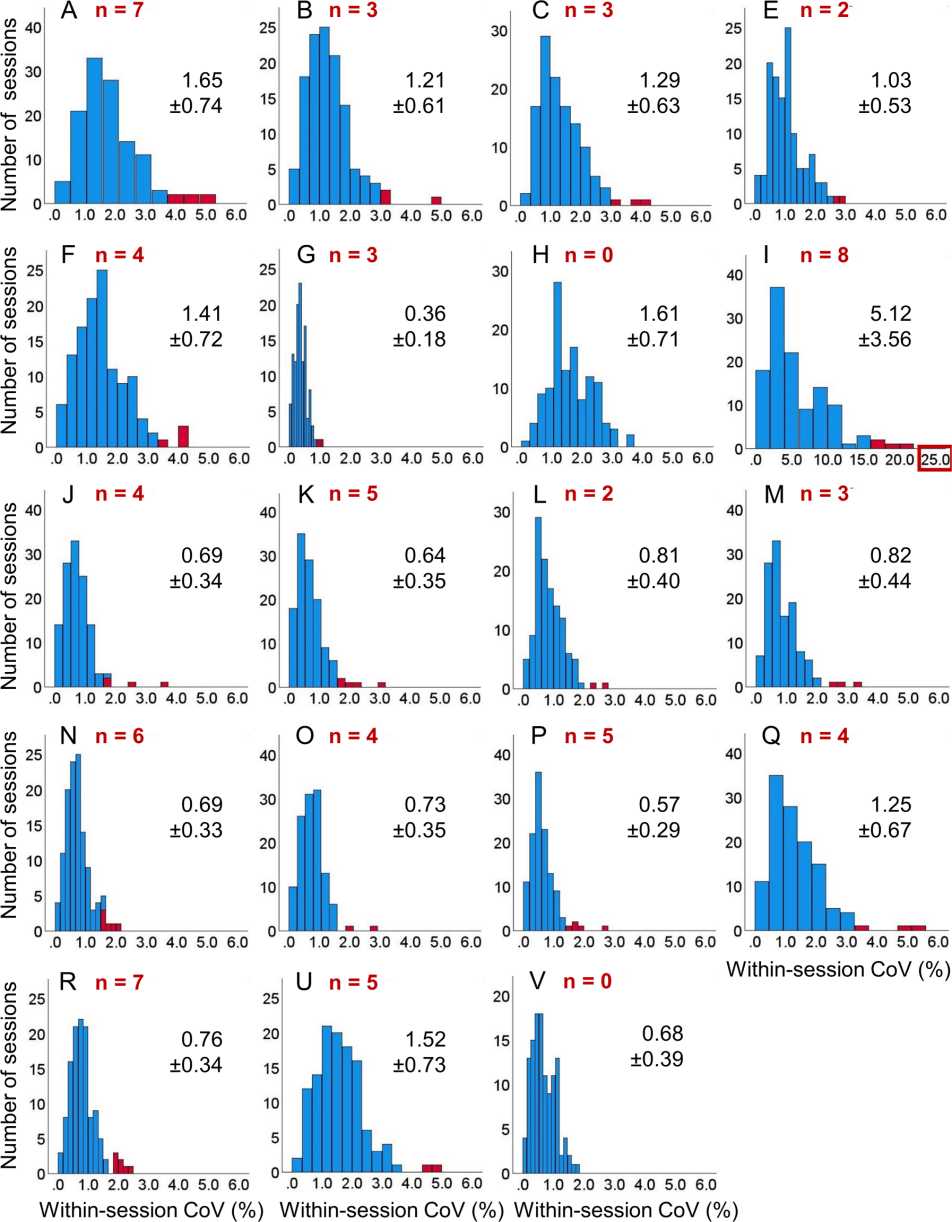
Within-session coefficient of variation (CoV) of hippocampus volume (HV) estimates in the left hemisphere: histogram of the within-session CoV (=100 × standard deviation / mean across all back-to-back repeat scans in the session) of the HV in the left hemisphere across the 122 included scanning sessions, separately for each volumetry tool (A, B, …, V). The (number of) outliers is shown in red color. Mean ± standard deviation of the CoV estimates were computed across the non-outlier sessions (blue). The scale of the horizontal axis is the same for all volumetry tools except tool I. As a consequence, not all outliers are displayed for all tools. Histograms of the HV within-session CoV in the right hemisphere are shown in Supplemental Figure 2.

The distribution of the within-session CoV of the left HV across the 122 included scanning sessions is shown in Figure 1, separately for each volumetry tool. The corresponding distributions of the within-session CoV of the right HV are shown in Supplemental Figure 2. The distributions of the within-session *mean* of left and right HV across the 122 scanning sessions are shown in Supplemental Figures 3 and 4, respectively.

**Figure 2.**
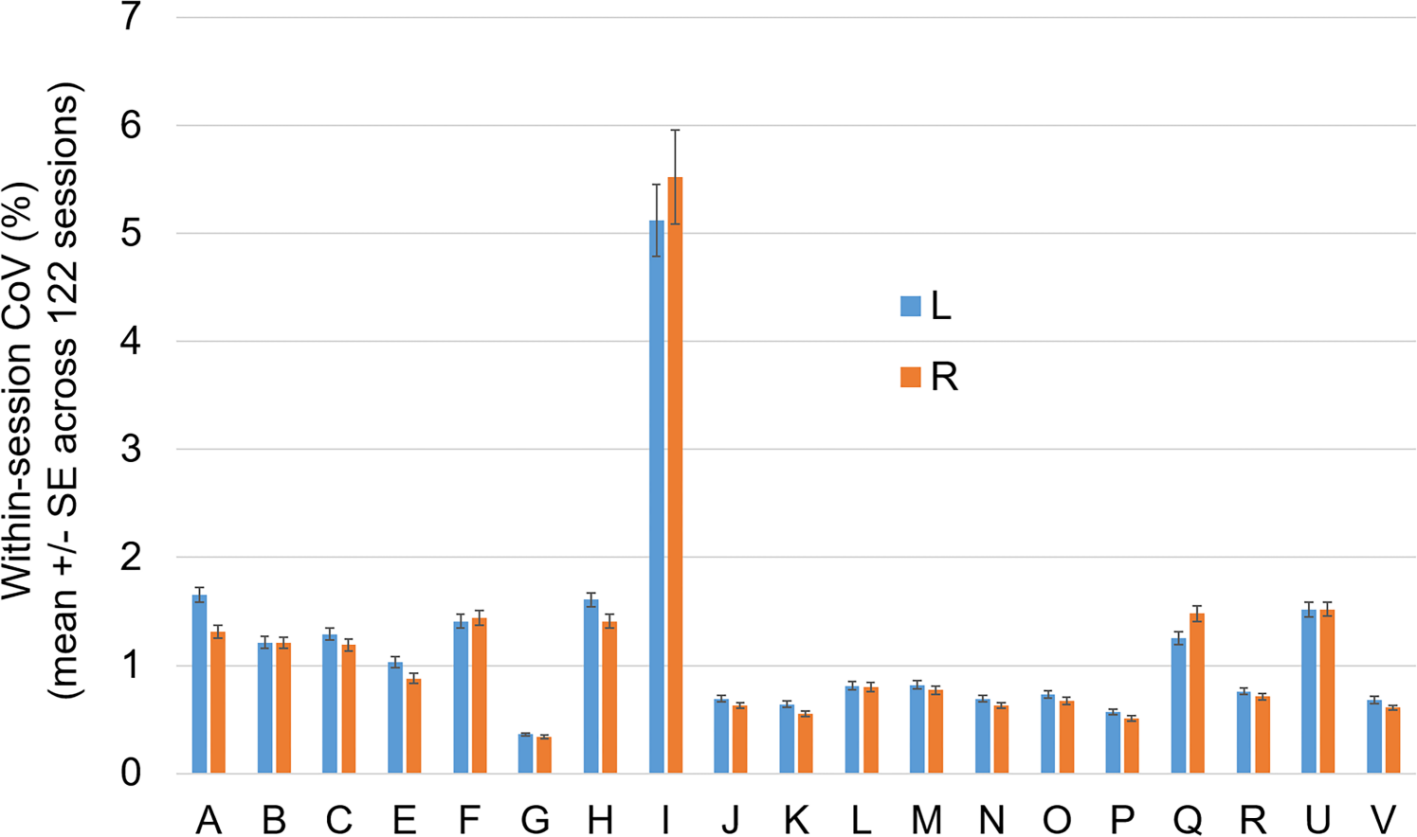
Mean within-session coefficient of variation (CoV): mean value ± standard error of the mean (SE) of the within-session CoV across all non-outlier scanning sessions, separately for each volumetry tool (A, B, …, V) and both hemispheres (L = left, R = right).

The mean value of the within-session CoV across all non-outlier scanning sessions is shown in Figure 2, separately for each volumetry tool. Volumetry tool I showed considerably higher within-session variability compared with all other tools and, therefore, was excluded from the computation of the mean value of the within-session CoV z-score across volumetry tools.

**Figure 3.**
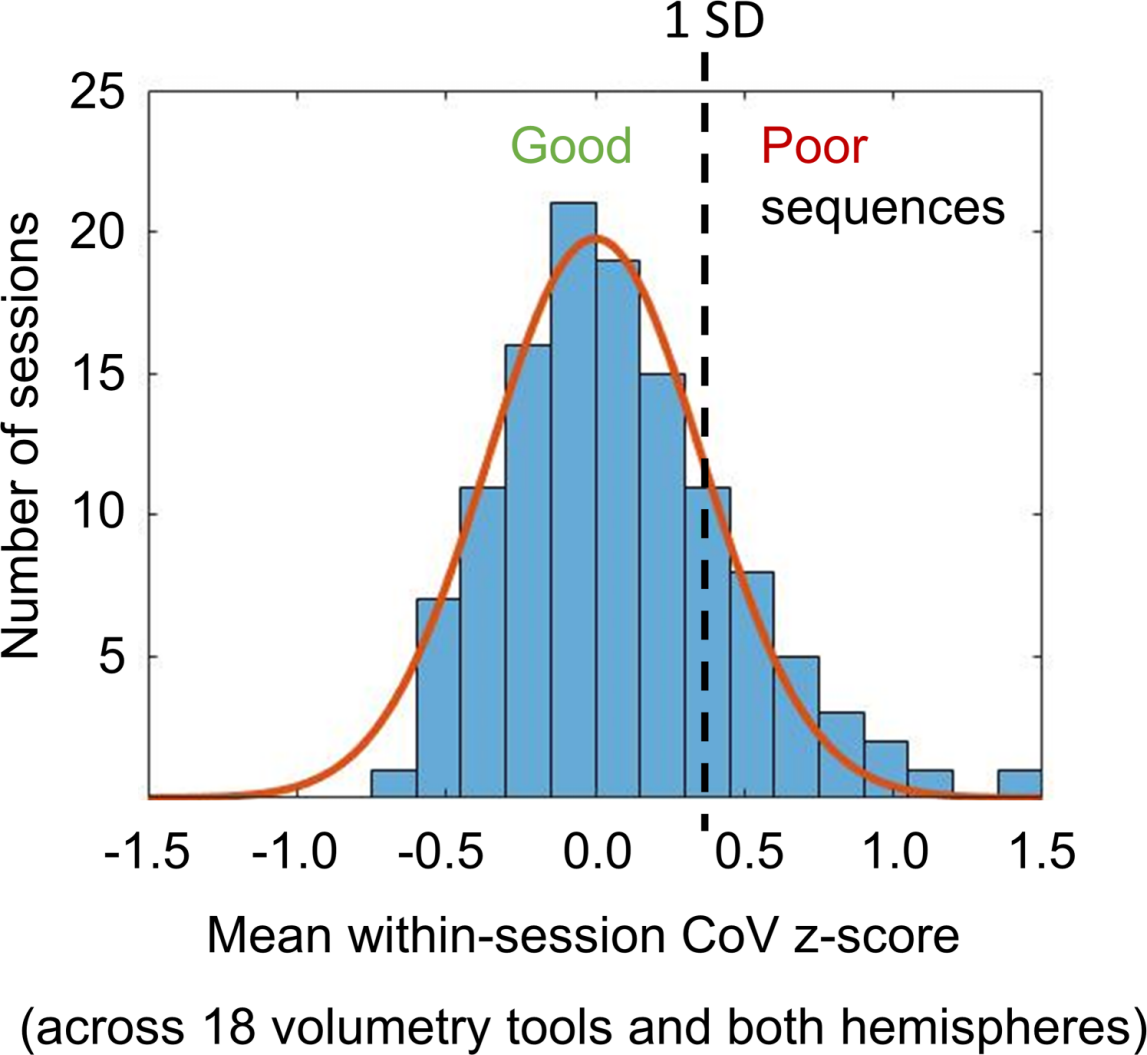
Mean value of the within-session CoV z-score across all volumetry tools (except tool I) and both hemispheres: the histogram shows the distribution of the mean z-score across the 122 scanning sessions. The continuous line represents the fit of a Gaussian function with its mean value fixed at z-score = 0. The standard deviation (SD = 0.360) of the Gaussian fit was used to distinguish between “good” acquisition sequences (high within-session stability of HV estimates on average across all volumetry tools) and “poor” acquisition sequences (low within-session stability): “good” if z-score ≤ + 1 SD, “poor” if z-score > + 1 SD.

The distribution of the mean (across the remaining 18 volumetry tools and both hemispheres) CoV z-score across the 122 scanning sessions is shown in Figure 3. The Gaussian fit of the distribution revealed a SD of 0.360 z-score points. Using +1.0 SD as cutoff on the mean CoV z-score identified 95 (77.9%) acquisition sequences to be good and 27 (22.1%) to be poor (Supplemental Figure 5).

The number of back-to-back repeat scans within a session did not differ between good and poor acquisition sequences (3.6 ± 0.7 versus 3.7 ± 0.8, t-test p = 0.707). There was a trend towards a higher proportion of poor acquisition sequences at ≤1.5T compared with 3T (26.1% versus 11.8%, Pearson chi-square p = 0.086). The proportion of poor acquisition sequences did not depend on the scan orientation (sagittal versus other, Pearson chi-square p = 0.219). Good and poor acquisition sequences did not differ with respect to the duration of a single scan (279 ± 59 s versus 296 ± 96 s, heteroscedastic t-test p = 0.398).

**Figure 4.**
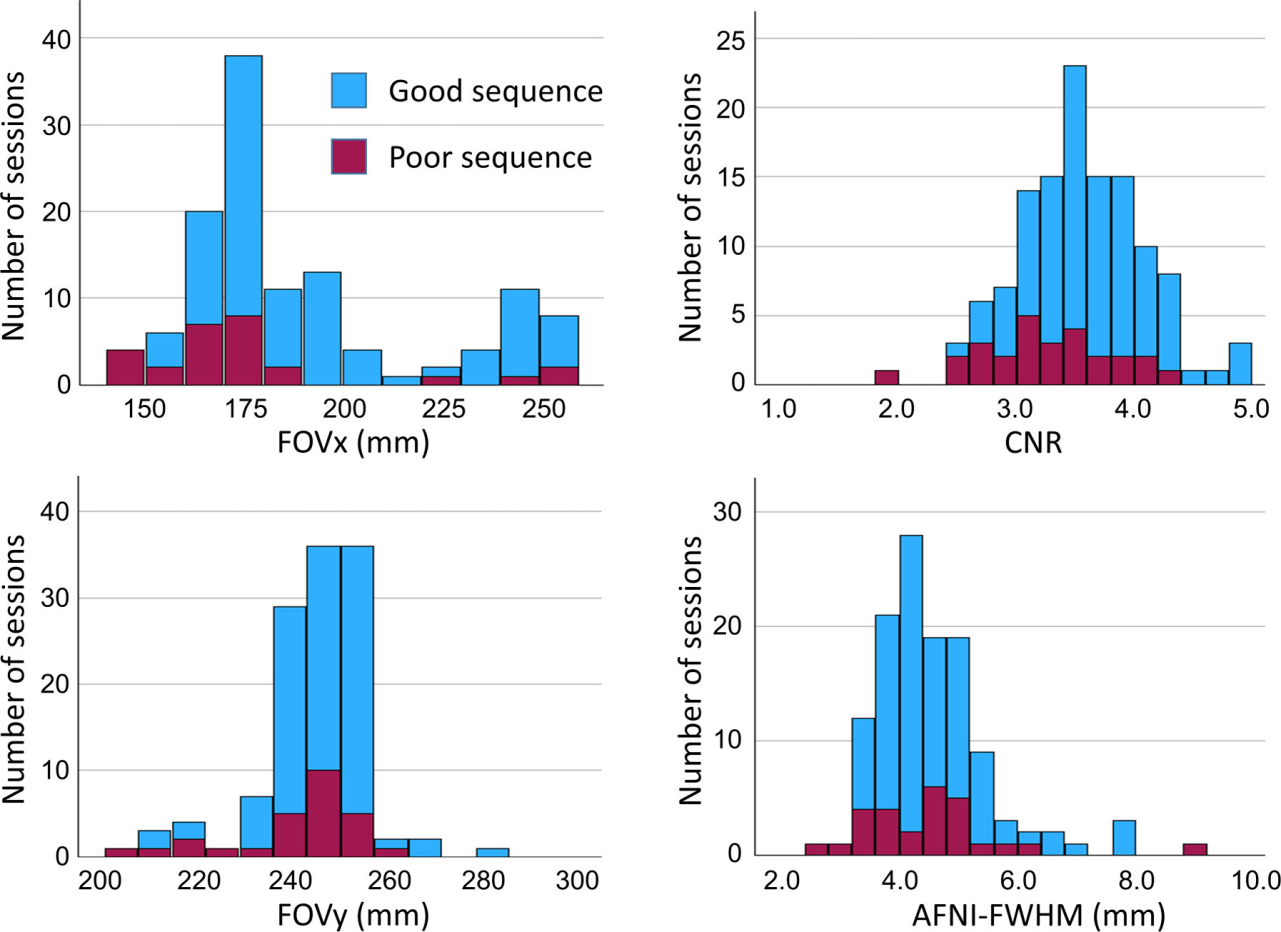
Image quality metrics: histograms of the acquisition field-of-view in left-right and anterior-posterior direction (FOVx, FOVy) and the session means of the MRIQC CNR and AFNI-FWHM across the 122 scanning sessions. “Good” and “poor” acquisition sequences are indicated by different colors.

Histograms of the 4 IQMs (FOVx, FOYy, CNR, AFNI-FWHM) across the 122 scanning sessions are shown in Figure 4.

**Figure 5.**
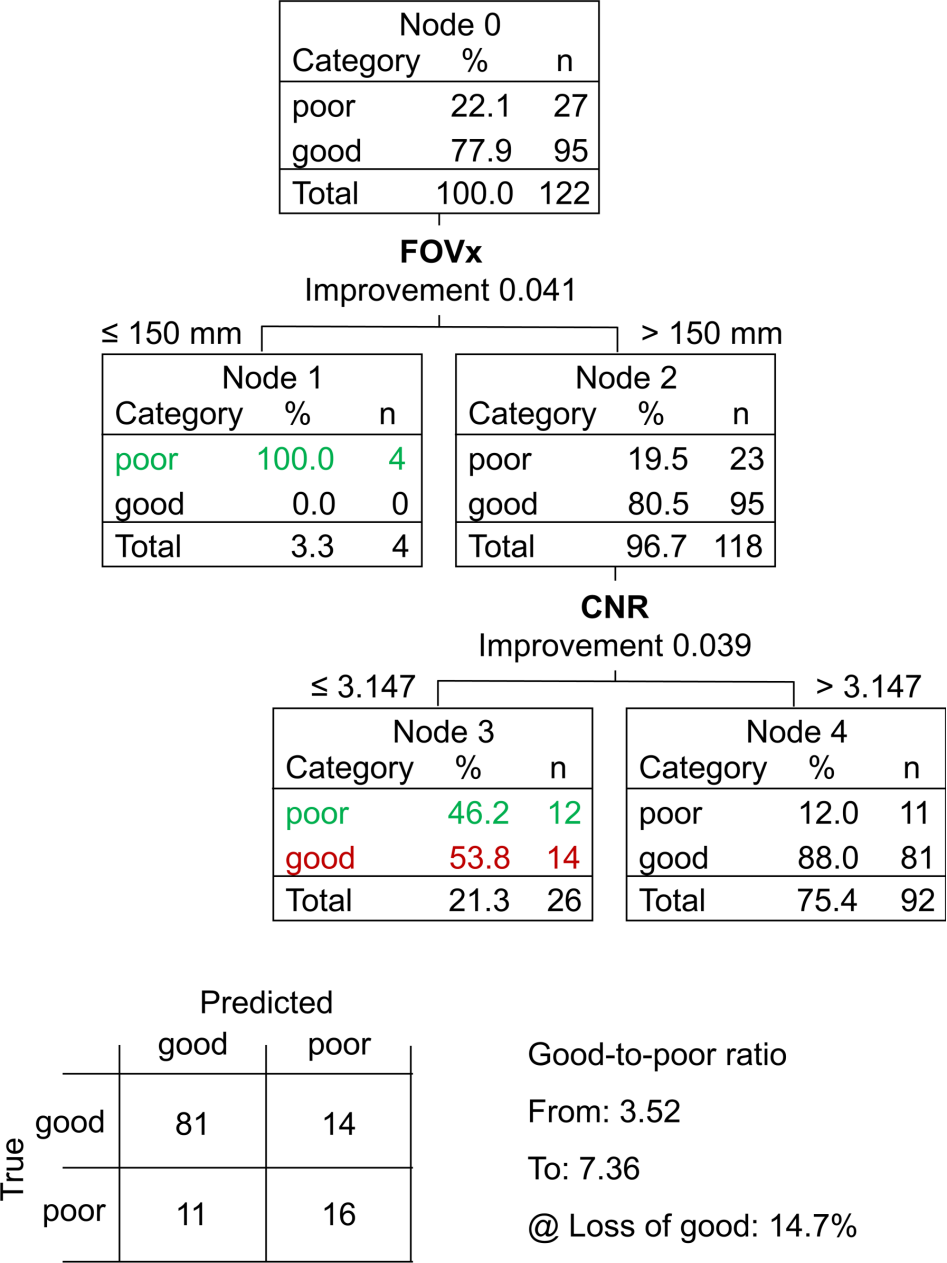
Classification and regression tree (CART) to discriminate between “poor” and “good” acquisition sequences: the field-of-view in left-right direction (FOVx) and the contrast-to-noise ratio (CNR) were included in the model. The field-of-view in anterior-posterior direction and the AFNI full-width-at-half-maximum were not included. Correctly identified “poor” acquisition sequences are shown in green, “good” acquisition sequences falsely classified as “poor” are shown in red. The 2 × 2 contingency tables of the CART prediction performance is shown at the bottom.

**Figure 6.**
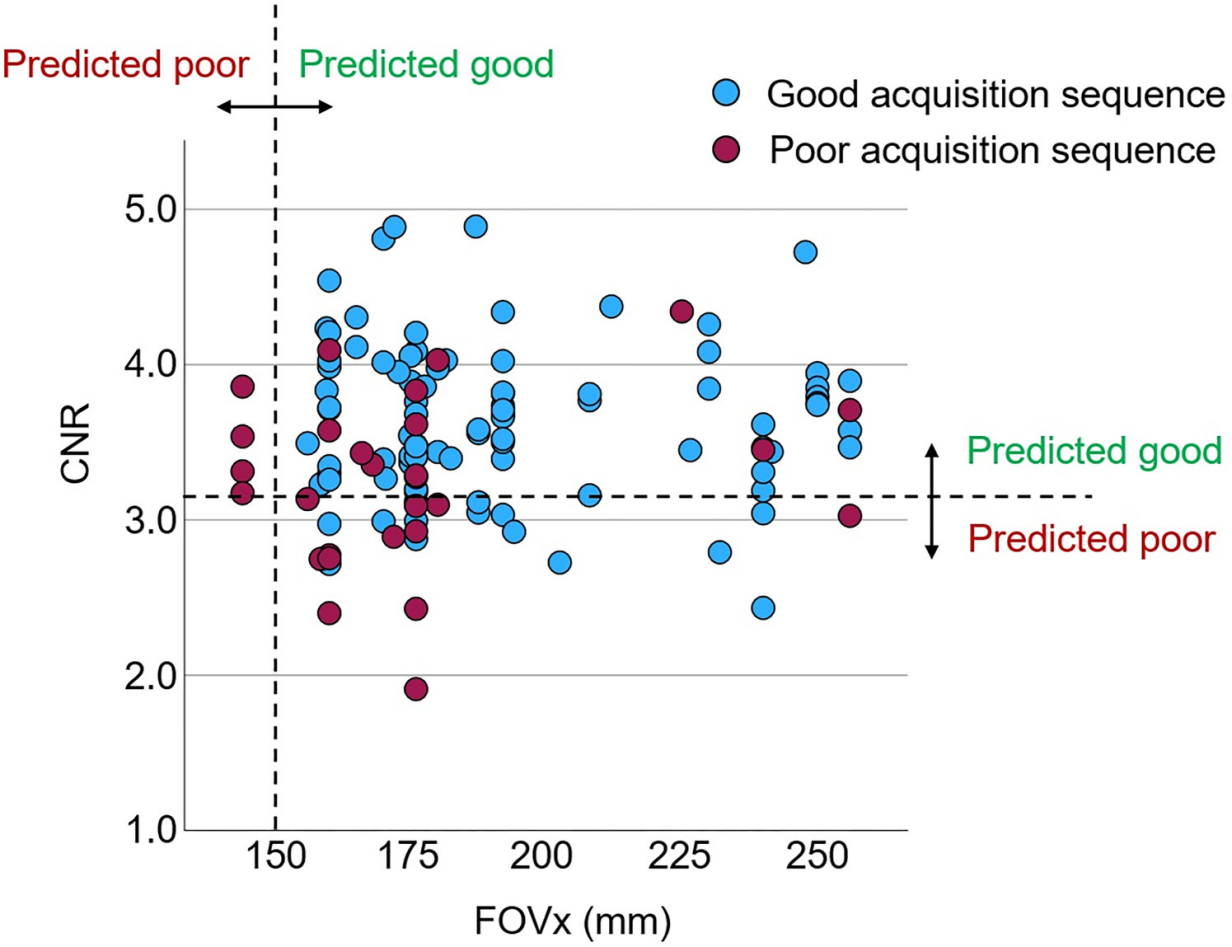
Performance of the classification and regression tree (CART) to discriminate between good and poor acquisition sequences: scatter plot of the within-session mean of the MRIQC contrast-to-noise ratio (CNR) versus the field-of-view in left-right direction (FOVx) across the 122 scanning sessions. The dashed lines represent the cutoffs on FOVx and CNR according to the CART (Figure 5).

**Figure 7.**
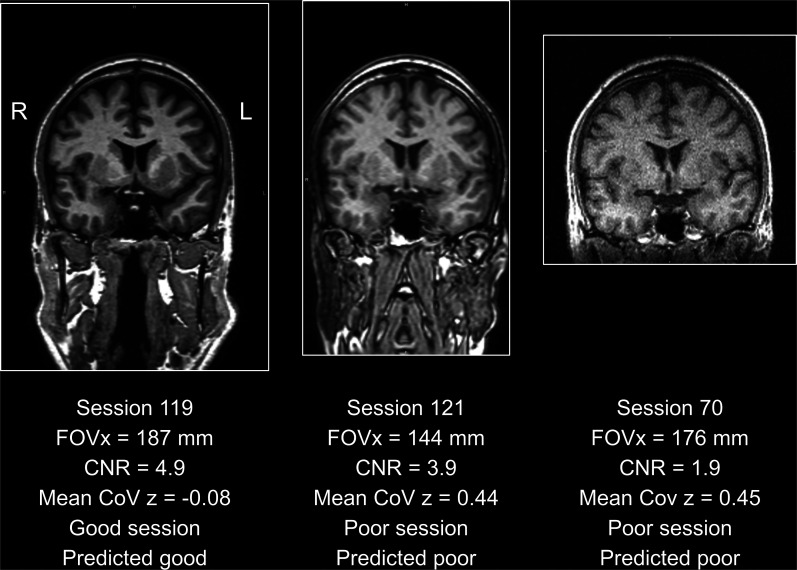
Representative acquisition sequences: the acquisition sequence used in scanning session 119 (left) passed the quality control by the classification and regression tree (CART, Figure 5): the field-of-view in left-right direction (FOVx) was above the cutoff of 150 mm on FOVx and the contrast-to-noise ratio (CNR) was above the cutoff 3.147 on the CNR. The within-session coefficient of variation (CoV) z-score (mean across 18 volumetry tools and both hemispheres) was negative, indicating better than average within-session test-retest stability of HV estimates. The acquisition sequence used in scanning session 121 (middle) was characterized by good CNR but poor FOVx. Thus, it was categorized as poor by the CART, in line with the mean within-session CoV of 0.44 clearly above the threshold of 0.36 (Figure 3). The acquisition sequence used in scanning session 70 (right) was acquired with an acceptable FOVx, but the CNR was clearly below the cutoff. Thus, it was categorized as poor, in line with the large mean within-session CoV above the threshold. For each session, the first scan is shown.

The CART to discriminate between “poor” and “good” acquisition sequences is shown in Figure 5. The FOVx was selected for the first branching, the CNR was selected for a second branching of the cases with adequate FOVx. FOVy and AFNI-FWHM were not included in the model. Overall accuracy, sensitivity and specificity of the CART for the identification of good sessions were 79.5%, 85.3% and 59.2%, respectively (Figure 6). CART-based classification increased the ratio of good-to-poor acquisition sequences from 3.5 among all sequences to 7.4 among the sequences predicted to be good. This was at the expense of losing 15% of the good acquisition sequences. Representative cases are shown in Figure 7.

**Figure 8.**
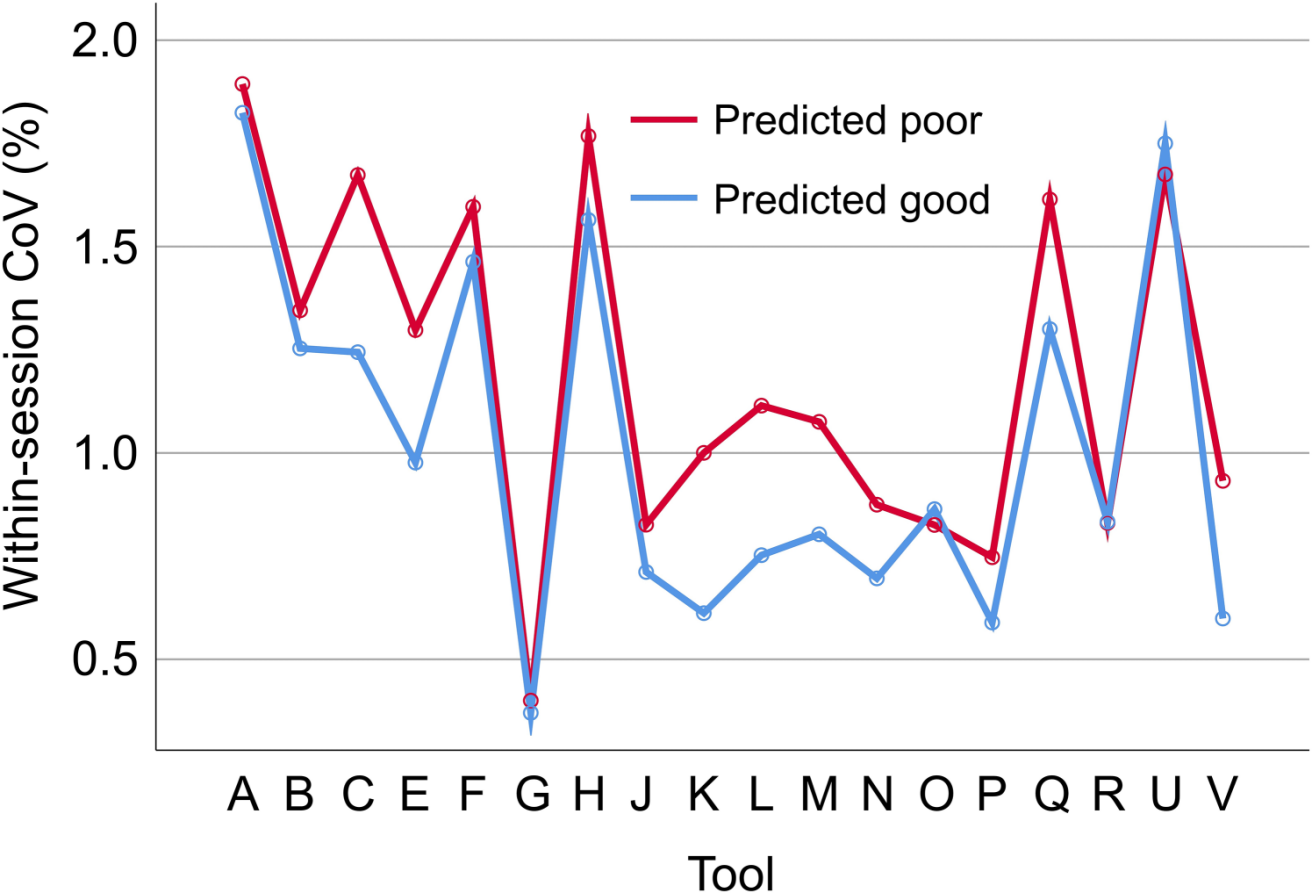
Impact of the classification and regression tree (CART)-based categorization on within-session test-retest stability of hippocampus volume estimates. Within-session coefficient of variation (CoV) of the volume estimates of the left hippocampus in scanning sessions categorized as good by the CART (mean CoV across all good sessions) versus scanning sessions categorized as poor by the CART (mean CoV across all poor sessions), separately for each of the 18 included volumetry tools. The corresponding results for the right hippocampus volume are shown in Supplemental Figure 6.

The repeated measures ANOVA of the within-session CoV revealed the impact of the volumetry tool to be highly significant (p < 0.001), whereas the tool * CART-based categorization interaction effect did not reach statistical significance (p = 0.595). The estimated marginal mean of the within-session CoV was 0.987% (standard error 0.022%) for acquisition sequences categorized as good by the CART versus 1.134% (0.038%) for acquisition sequences categorized as poor by the CART. The within-session CoV was smaller for acquisition sequences categorized as good by the CART compared with the acquisition sequences categorized as poor for 16 (88.9%) of the 18 included volumetry tools (Figure 8). For the remaining 2 (11.1%) volumetry tools, the within-session CoV was slightly larger for acquisition sequences predicted to be good. For the excluded volumetry tool I, the within-session CoV was significantly smaller for acquisition sequences categorized as good by the CART compared with the acquisition sequences categorized as poor in both hemispheres (left: 5.3% versus 12.3%, p = 0.038; right: 5.7% versus 14.1%, p = 0.015).

## Discussion

The primary finding of this study was that a simple CART model for binary classification of acquisition sequences based on only two general IQM (estimated from human phantom scans acquired with the considered acquisition sequences) provides useful accuracy for the discrimination between acquisition sequences with good versus poor (within-session) test-retest stability of HV estimates (Figures 5 and 6). This was relatively independent of the volumetry tool (Figure 8), suggesting that the same CART is beneficial with different volumetry tools.

A typical application scenario is as follows. For a given acquisition sequence at a given MR scanner, the freely available MRIQC tool would be used to obtain FOVx and CNR from human phantom scans acquired in this setting. If the CART predicts the acquisition sequence to be poor with respect to within-session stability of hippocampus volumetry, the acquisition parameters of the sequence should be adjusted, particularly with the aim of improving FOVx and/or CNR. We hypothesize that this will improve the stability of hippocampus volumetry in clinical routine across sites and scanners.

### IQM

From the whole set of 68 IQM provided by the MRIQC tool, only two were considered for the discrimination between good and poor acquisition sequences with respect to test-retest stability of hippocampus volumetry, CNR and AFNI-FWHM, because these were expected to be particularly useful for this task.^33,56,79^ The aim was to keep the prediction model not only easy-to-use but also easy-to-interpret by avoiding possible collinearity issues among a larger set of predictors. Collinearity does not affect the performance of the whole model but strongly limits its interpretability with respect to individual predictors. MRIQC IQM that may be particularly sensitive to specific characteristics of the individual human phantom's brain were not used. For example, MRIQC IQM that characterize the overlap of the tissue probability maps from the individual image with the corresponding normal tissue probability maps were disregarded for this reason. MRIQC signal-to-noise ratio metrics using the air background as a sole noise reference were also disregarded,^80^ since the noise characteristics of the air signal often is not a reliable measure of the image quality of the tissue segments.^75,81,82^ The coefficient of joint variation ( $\mathrm{CJV}=({\hat{\sigma }}_{GM}^{2}+{\hat{\sigma }}_{WM}^{2})/|{\hat{\mu }}_{GM}-{\hat{\mu }}_{WM}|$ , mriqc.readthedocs.io/en/latest/iqms/t1w.html)^83^ was disregarded, because it showed a very strong inverse correlation with the CNR (Spearman correlation coefficient of the session means across the 122 included sessions = −0.992, p < 0.001).

IQM and multi-variable IQM-based models for automatic or semi-automatic quality assessment of structural brain MRI have been proposed by various groups^26,79,84–88^ (systematic review^81^). Some of the volumetry tools used in the current study provide their own IQM. The rationale for using MRIQC IQM was that the MRIQC software is freely available (both for local implementation and as online service^29^) so that users can have access to these IQM independent of the volumetry tool they are using. Furthermore, IQM provided by a given volumetry tool may depend on that tool resulting in unwanted bias.

The proposed application scenario assumes that the variability of the IQM between sMRI scans of different human phantoms acquired with the same scanner and the same acquisition sequence is smaller than their difference between “poor” and “good” scanning sessions acquired with the same human phantom. Pouwels and co-workers assessed the between-site variability of MRIQC IQM in 3D T1-weighted scans between 5 different 3T scanners from 3 different manufacturers (Philips, GE, Siemens) at 5 different sites.^89^ The acquisition sequences had been previously harmonized between the sites according to ADNI-3 recommendations.^90^ At each site, 28 healthy subjects were selected and scanned at this site. The subjects were matched for age, sex and education between the sites. The mean (across the 5 sites) of the between-*subjects* CoV was smaller than the between-*sites* CoV of the site-specific mean values for each of the MRIQC IQM tested by Pouwels and co-workers.^89^ For the AFNI-FWHM, the mean (across the 5 sites) of the between-*subjects* CoV (3.05%) was about 3 times smaller than the between-*sites* CoV of the site-specific mean values (9.8%). For the CJV, the mean of the between-*subjects* CoV (7.5%) was about 1.4 times smaller than the between-*sites* CoV of the site-specific mean values (10.6%). A similar relationship can be assumed for the CNR due to its strong inverse correlation with the CJV (see above). This demonstrates that the variability of the MRIQC CNR IQM is smaller between different healthy subjects than between sites/scanners even in case of strict harmonization of the T1 acquisition sequences. Thus, it can be safely assumed that the variability of these IQM between different human phantoms is much smaller than the difference between “poor” and “good” acquisition sequences in non-harmonized settings.

We hypothesize that the exclusion of poor acquisition sequences is useful also for retrospective multi-site harmonization of sMRI scans, either using conventional approaches such as ComBat^91^ or deep learning-based methods such as SynthSR,^92^ MURD^18^ or CALAMITI.^93^ Retrospective image harmonization may not only be less effective for poor acquisition sequences, but including poor acquisition sequences in the procedure may affect the model's performance also in good sequences.

### Volumetry tools

A secondary finding of this study was the rather large difference between volumetry tools regarding the within-session stability of HV estimates. Among the 19 considered volumetry tools, the average of the HV within-session CoV across all non-outlier scanning sessions ranged between 0.3 and 5.5% (Figure 2).

Another secondary finding was the rather large difference between the volumetry tools regarding their sensitivity with respect to the acquisition sequence.^17,20^ The *between*-sessions CoV of the within-session mean of the HV estimates varied between about 1% and almost 7% (Supplemental Figure 7). The tight correlation of the between-sessions CoV of left and right hemisphere (Supplemental Figure 7) suggests that the between-sessions CoV was mainly driven by differences in the image characteristics between the scanners and acquisition protocols used for the different scanning sessions. In multi-site/multi-scanner settings, volumetry tools with low between-sessions CoV may be preferred.

A third secondary finding was the rather large difference of HV estimates between different volumetry tools, in line with previous studies.^94^ Among the 18 volumetry tools that provide HV estimates in ml (all except tool G), the average of the HV within-session mean across all non-outlier scanning sessions ranged between 2.15 and 5.99 ml for the left hemisphere and between 1.93 and 5.55 ml for the right hemisphere (Supplemental Figures 3 and 4). These differences are largely explained by different anatomical hippocampus definitions (e.g., with or without subiculum). The latter prompted an international group of experts to propose a harmonized protocol for the segmentation of the hippocampus on MR images both manually^95,96^ and with software tools for fully automatic segmentation.^97^ The fact that not all tools for automatic HV estimation are in accordance with this recommendation complicates the interpretation of HV estimates, not only because it limits the utility of longitudinal HV estimates obtained with different volumetry tools, but also because age-related decline and disease-related atrophy differ between hippocampal subregions.^98,99^

### Limitations

Limitations of the current study include the following. First, a CART and only four general IQM were tested for the discrimination between good and poor acquisition sequences with respect to test-retest stability of hippocampus volumetry. More complex classification methods often provide better performance compared to low-dimensional decision trees, but at the expense of lower transparency (“black-box” methods).^100^ Low-dimensional decision tree models allow the user to verify that the classification decision made by the algorithm is plausible and coherent, which improves their acceptance for widespread clinical use.^101^ The CART variant among decision tree models uses a binary split at each node, which further simplifies the interpretation.

Second, the within-session CoV z-score averaged across all volumetry tools (and both hemispheres) was used for the definition of good versus poor acquisition sequences. Only one of the 19 considered volumetry tools was excluded, because it resulted in 3 to 16 times higher within-session variability compared with all other tools (Figure 2). The rationale for averaging over different volumetry tools was to generate a model for the classification of acquisition sequences that is independent of a specific volumetry tool. However, it is expected that a model trained for a specific volumetry tool can provide greater improvement of test-retest stability of HV estimates compared with the generic CART proposed here. The added value most likely is most pronounced for those volumetry tools that did not benefit from the generic CART (Figure 8, Supplemental Figure 6). All included volumetry tools have been validated technically and/or clinically for use in clinical practice and/or research (although the corresponding data may not have been published for each tool^1^).

Third, test-retest stability of HV estimates was characterized by the CoV z-score across consecutive back-to-back repeat scans that were acquired without delay and without repositioning during a single scanning session. The FTHP dataset does not allow testing test-retest stability across scans with repositioning of the head.

Fourth, short-term test-retest stability of HV estimates across back-to-back repeat scans in the same scanning session may be improved by longitudinal processing.^102,103^ This could not be tested here, as the participating developers/providers of the volumetry tools were blinded with respect to the scanning session.

Fifth, the threshold on the CoV z-score for the discrimination between “good” and “poor” scanning sessions was derived from the distribution of the mean within-session CoV z-score across all volumetry tools, that is, from the same data to which it then was applied. This approach will always identify “poor” sessions, even if all considered sequences are actually adequate. In the latter case, the “poor” sequences are not really bad but just “worse” than the “good” sequences.

Sixth, the study was blinded with respect to the volumetry tools. The developers/providers of the volumetry tools had been assured that the identity of the volumetry tools is not disclosed to avoid potential conflicts of interest, particularly among the commercial participants. However, we do not consider this a major limitation of the study, since the FTHP dataset does not allow direct comparison of volumetry tools with respect to clinically relevant tasks, e.g., detection of Alzheimer's disease or prediction of progression from mild cognitive impairment to dementia. Furthermore, the findings regarding within-session and between-sessions variability of HV estimates do not allow reliable conclusions regarding the performance of the tools in these clinical tasks, because volumetry tools that have the strongest inbuilt biases are likely to appear to be the most stable (a tool that simply returns a value of 3.0 ml for all hippocampi is perfectly stable but it is clearly useless). Most volumetry tools involve some kind of regularization, and increasing the amount of regularization will reduce variance but often at the expense of increasing bias.

Seventh, the decision tree model was derived from the same dataset on which it was tested. This might have resulted in overly optimistic performance estimates due to overfitting, despite the use of 5-fold cross-validation. Thus, the proposed decision tree (including its thresholds) requires validation in independent (out-of-distribution) test datasets.

Eighth, this study had an exclusive focus on test-retest stability of hippocampus volumetry. Thus, the decision tree model may not be transferred to MRI-based volumetry of other brain regions without adjustment.

Finally, motion artefacts present a significant problem in clinical practice and also cannot be ruled out in human phantom scans. This could result in the erroneous categorization of an acquisition sequence as “good” or as “poor” with respect to test-retest stability of hippocampus volumetry. Therefore, it is crucial to strictly avoid motion in human phantom scans intended for characterizing the acquisition sequence used (as implied by the term “phantom”). The IQM employed in the current study to train the CART model (CNR, AFNI-FWHM, FOVx, FOVy) are not particularly sensitive to motion and are therefore not useful for detecting or excluding (subtle) motion. Of the general IQM provided by the MRIQC software package, the entropy-focus criterion (EFC), which is based on the Shannon entropy of the voxel intensities, is sensitive to ghosting and blurring induced by head motion.^29^ Thus, the following two-step approach could be used to characterise an acquisition sequence as either “good” or “poor” with respect to test-retest stability in hippocampus volumetry based on human phantom scans: First, the EFC is used to check for relevant motion artefacts in the given human phantom scans. If the scans pass this first step, indicating that there are no relevant motion artefacts, the proposed CART model can be used in the second step to classify the acquisition sequence with respect to test-retest stability in hippocampus volumetry. If the human phantom scans do not pass the first step, indicating relevant motion, human phantom scanning should be repeated using the same scanner with the same acquisition sequence. In the current study, the EFC (within-session mean) did not differ significantly between the 95 “good” scanning sessions and the 27 “poor” scanning sessions when assessed using the heteroscedastic independent samples t-test (EFC = 0.601 ± 0.085 versus EFC = 0.615 ± 0.063, p = 0.358). This suggests that motion was not a major factor in discriminating between “good” and “poor” scanning sessions in the FTHP dataset. This was to be expected, given that the healthy man who served as the human phantom for the FTHP dataset was particularly motivated to avoid any head motion during the MR scans.

In conclusion, an easy-to-use and easy-to-interpret IQM-based decision tree model provides useful performance for the discrimination between T1-weighted sequences associated with good versus poor test-retest stability of hippocampus volumetry. A narrow field-of-view in left-right direction (<150 mm) and low contrast-to-noise ratio were identified as risk factors for low test-retest stability of HV volumetry. This might be useful to increase the statistical power for the detection of cross-sectional differences or longitudinal changes in large-scale multicenter hippocampal volumetry research.

## Supplemental Material

sj-docx-1-alz-10.1177_13872877251380301 - Supplemental material for Easy-to-use and easy-to-interpret quality control of 3D gradient echo T1-weighted MR acquisition sequences for improved test-retest stability of MRI-based hippocampus volumetry Supplemental material, sj-docx-1-alz-10.1177_13872877251380301 for Easy-to-use and easy-to-interpret quality control of 3D gradient echo T1-weighted MR acquisition sequences for improved test-retest stability of MRI-based hippocampus volumetry by Ralph Buchert, Per Suppa, Babak A Ardekani, Fuensanta Bellvís Bataller, Pierrick Bourgeat, Pierrick Coupé, Robert Dahnke, Gabriel A Devenyi, Simon Fristed Eskildsen, Clara Fischer, Jose Vincente Manjón Herrera, Christian Ledig, Andreas Lemke, Bénédicte Maréchal, Roland Opfer, Diana M Sima, Lothar Spies, Aziz M Ulug and Hans-Jürgen Huppertz in Journal of Alzheimer's Disease
