# Supplementary material for: Easy-to-use and easy-to-interpret quality control of 3D gradient echo T1-weighted MR acquisition sequences for improved test-retest stability of MRI-based hippocampus volumetry
Source: J Alzheimers Dis. 2025 Sep 24;108(2):844–61. doi: 10.1177/13872877251380301 (PMC12605328; doi:10.1177/13872877251380301)
Supplement: sj-docx-1-alz-10.1177_13872877251380301 - Supplemental material for Easy-to-use and easy-to-interpret quality control of 3D gradient echo T1-weighted MR acquisition sequences for improved test-retest stability of MRI-based hippocampus volumetry [file sj-docx-1-alz-10.1177_13872877251380301.docx]

**Supplemental Material**

**Easy-to-use and easy-to-interpret quality control of 3D gradient echo T1-weighted MR acquisition sequences for improved test-retest stability of MRI-based hippocampus volumetry**

**
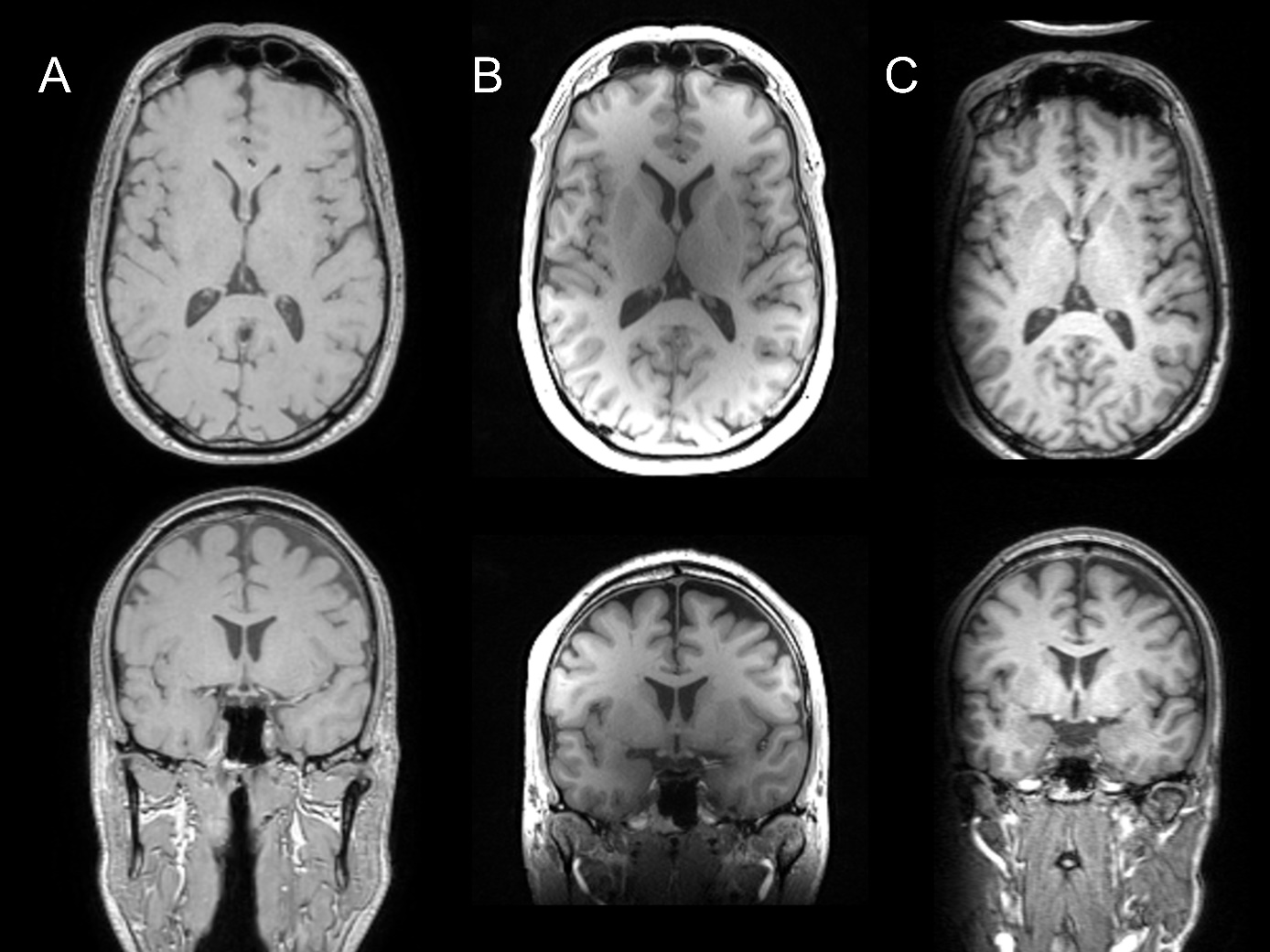
**

**Supplemental Figure 1 Representative scans excluded due to non-acceptable image quality according to visual inspection.** (A) Very low gray-to-white matter contrast, (B) severe bias field, or (C) too narrow field-of-view not including the entire head.

**
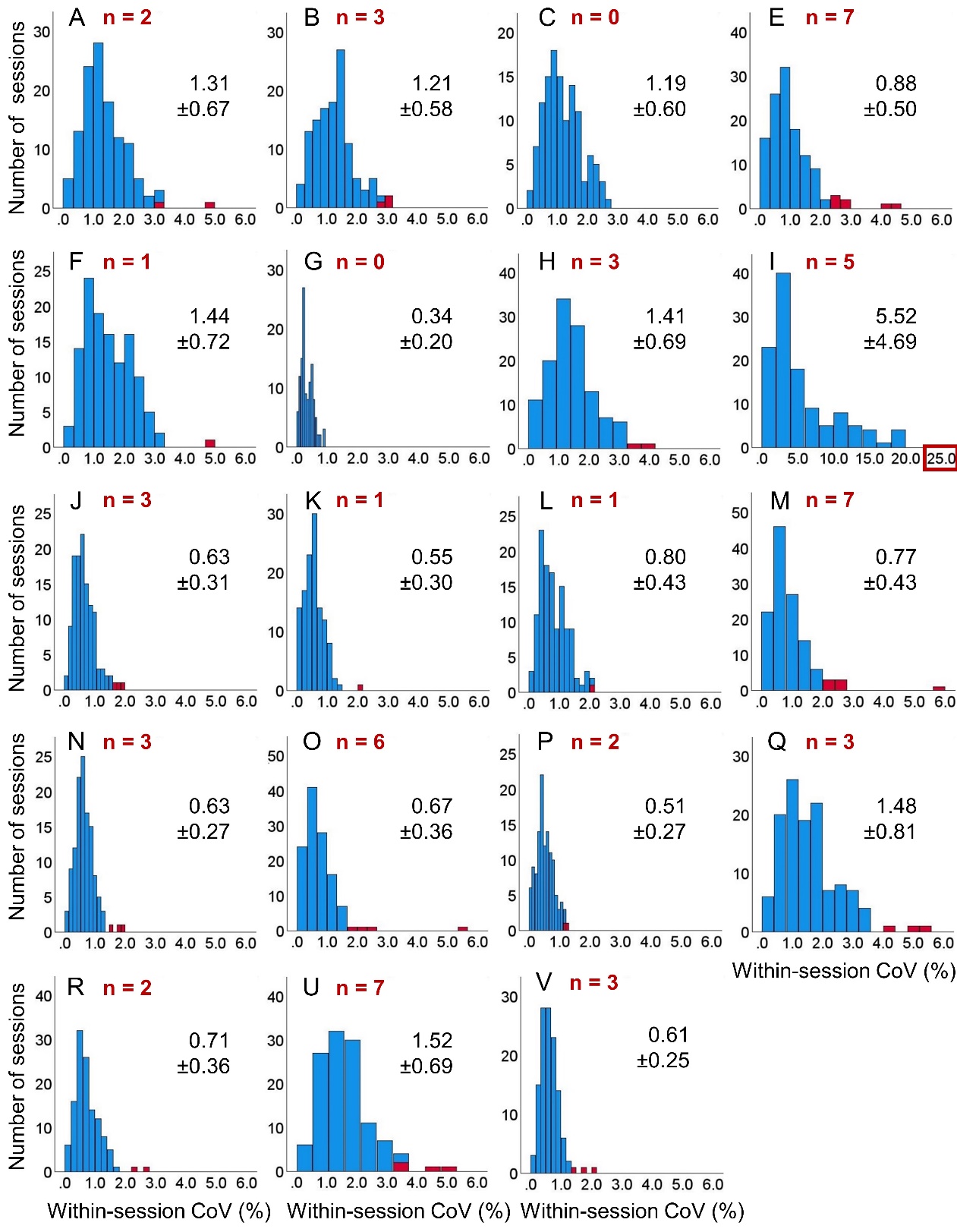
**

**Supplemental Figure 2.** **Within-session coefficient of variation (CoV) of hippocampus volume (HV) estimates in the right hemisphere.** Histogram of the within-session CoV (=100 * standard deviation / mean across all back-to-back repeat scans in the session) of the HV in the right hemisphere across the 122 included scanning sessions, separately for each volumetry tool (A, B,…, V). The (number of) outliers is shown in red color. Mean ± standard deviation of the CoV estimates were computed across the non-outlier sessions (blue). The scale of the horizontal axis is the same for all volumetry tools except tool I. As a consequence, not all outliers are displayed for all tools. Histograms of the HV within-session CoV in the left hemisphere are shown in Figure 1 in the manuscript.

**
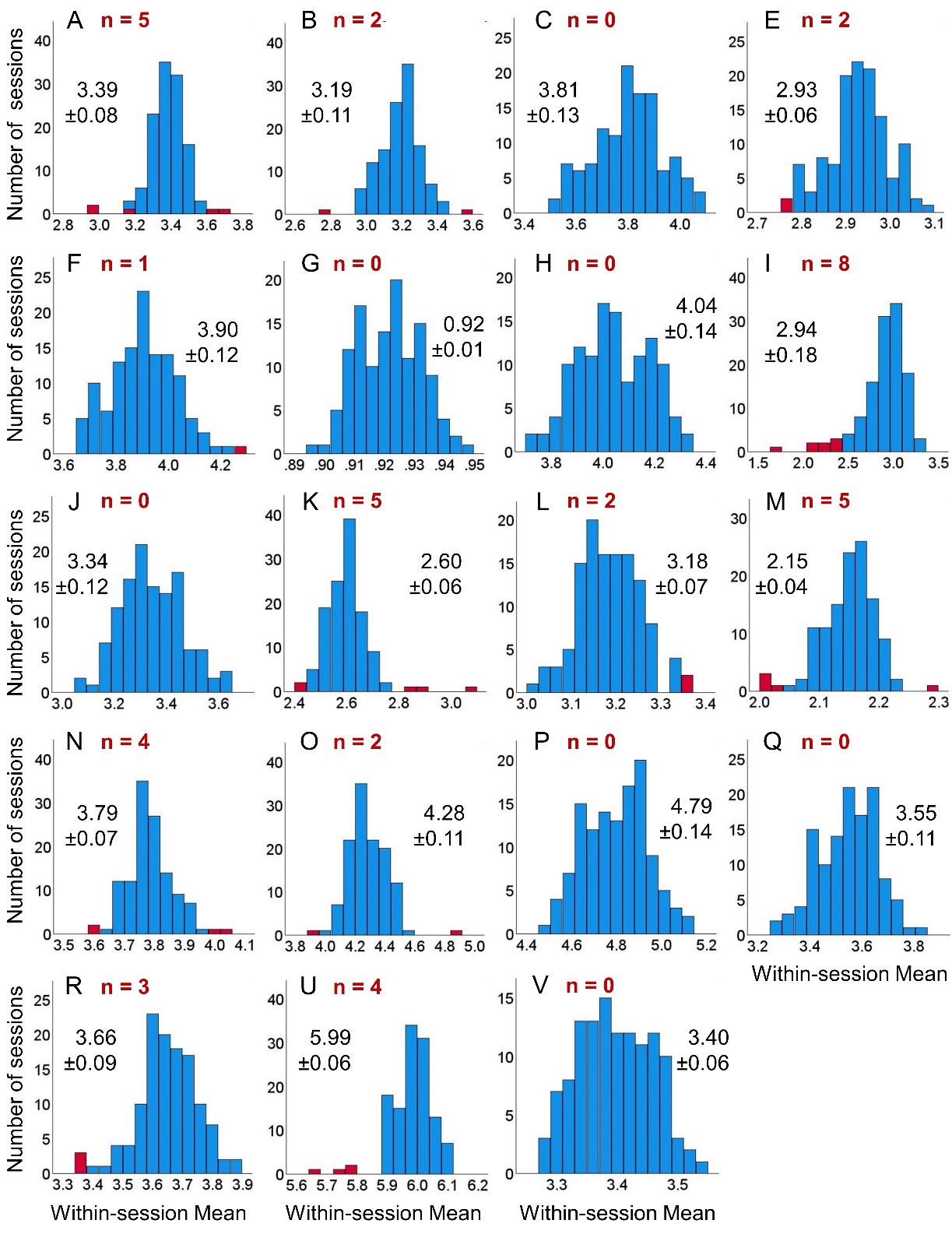
**

**Supplemental Figure 3.** **Within-session mean of hippocampus volume (HV) estimates in the left hemisphere.** Histogram of the within-session mean (mean value across all back-to-back repeat scans in the session) of the HV in the left hemisphere across the 122 different scanning sessions, separately for each volumetry tool (A, B,…, V). The (number of) outliers is shown in red color. Mean ± standard deviation of the HV estimates were computed across the non-outlier sessions (blue). HV estimates are in ml for all tools except for tool G that provides a hippocampal volumetric integrity index ranging between 0 and 1. The scale of the horizontal axis differs between the volumetry tools. Histograms of the HV within-session mean in the right hemisphere are shown in Supplemental Figure 4.

**
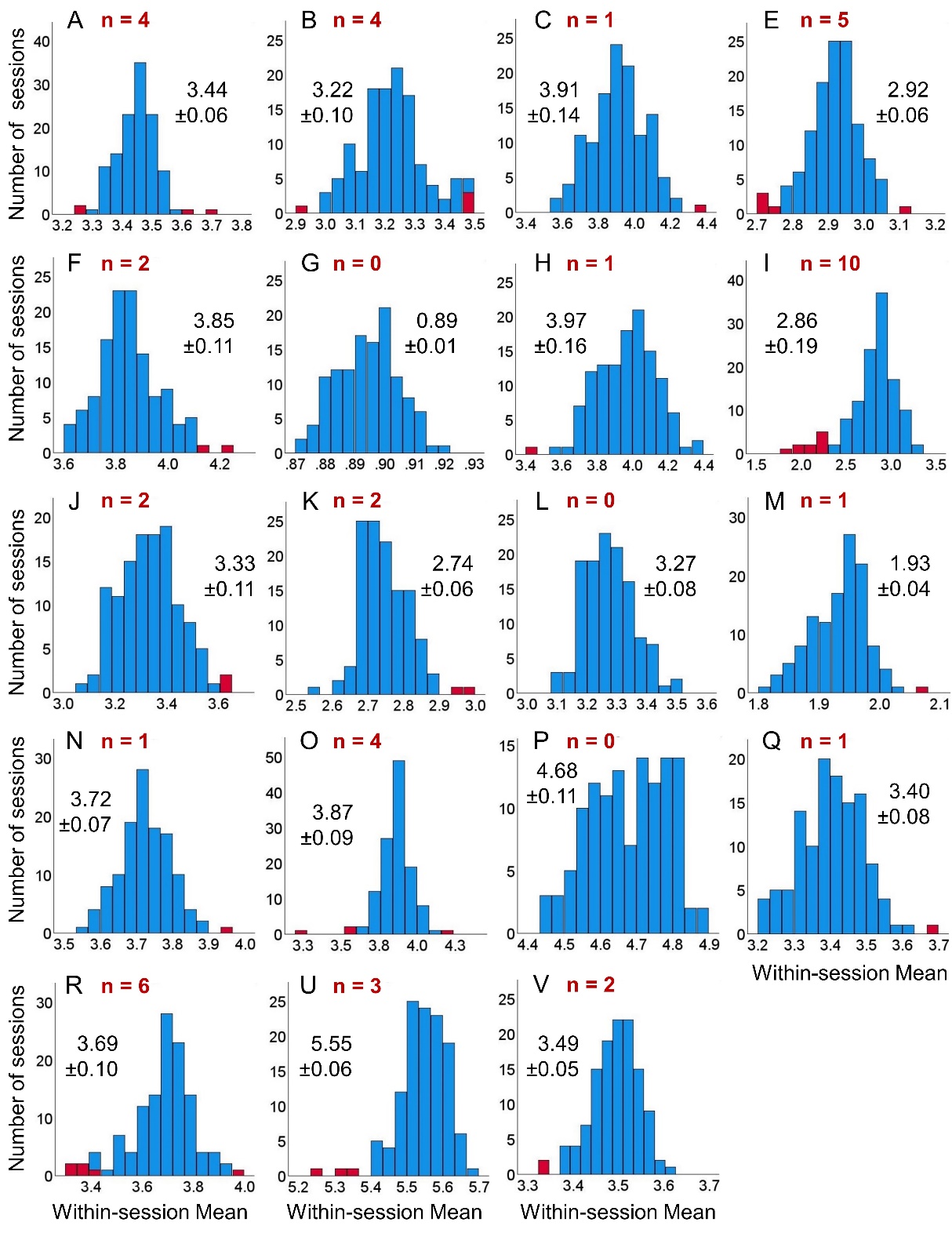
**

**Supplemental Figure 4.** **Within-session mean of hippocampus volume (HV) estimates in the right hemisphere.** Histogram of the within-session mean (mean value across all back-to-back repeat scans in the session) of the HV in the right hemisphere across the 122 different scanning sessions, separately for each volumetry tool (A, B,…, V). The (number of) outliers is shown in red color. Mean ± standard deviation of the HV estimates were computed across the non-outlier sessions (blue). HV estimates are in ml for all tools except for tool G that provides a hippocampal volumetric integrity index ranging between 0 and 1. The scale of the horizontal axis differs between the volumetry tools. Histograms of the HV within-session mean in the left hemisphere are shown in Supplemental Figure 3.


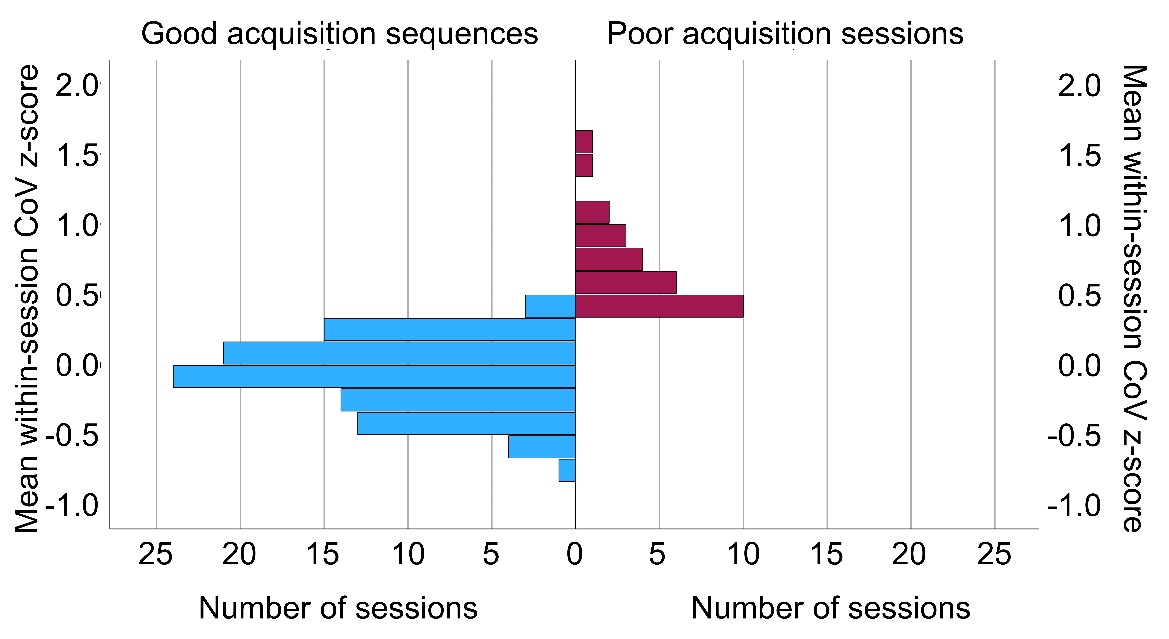


**Supplemental Figure 5.** **Mean (across 18 volumetry tools and both hemispheres) within-session CoV z-score.** The histograms show the distribution of the mean z-score separately for good and poor acquisition sequences.


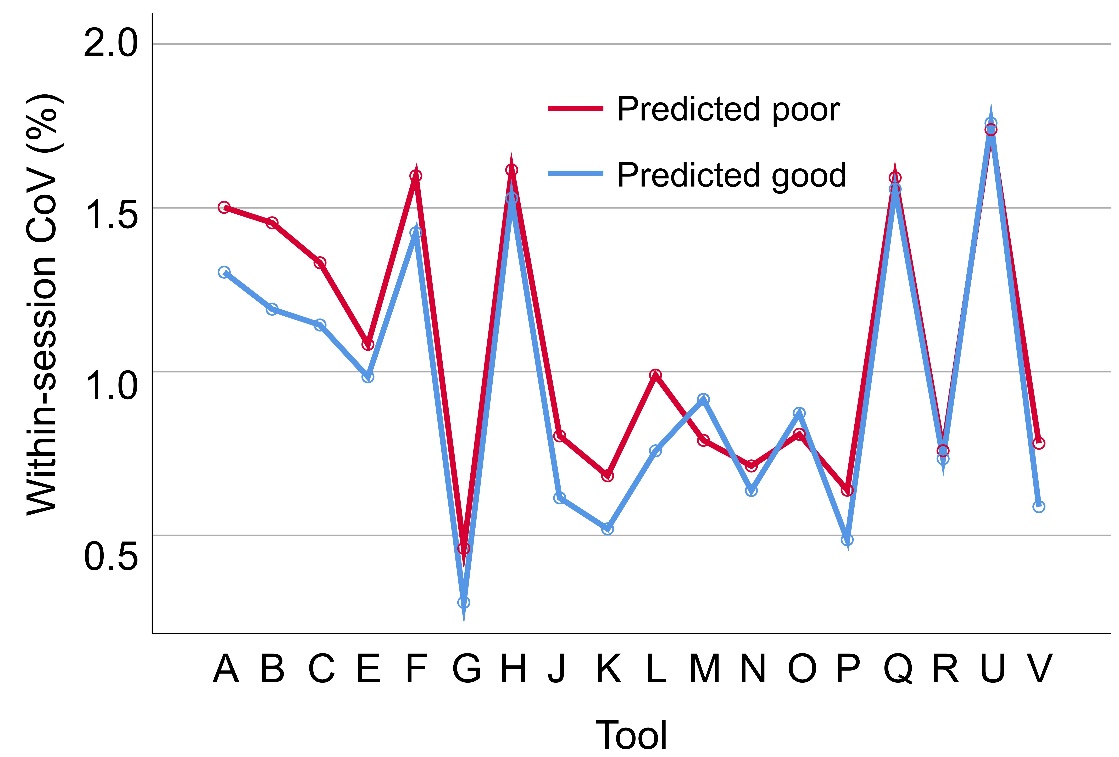


**Supplemental Figure 6.** **Impact of the classification and regression tree (CART)-based categorization on within-session test-retest stability of hippocampus volume estimates.** Within-session coefficient of variation (CoV) of the volume estimates of the right hippocampus in scanning sessions categorized as good by the CART (mean CoV across all good sessions) versus scanning sessions categorized as poor by the CART (mean CoV across all poor sessions), separately for each of the 18 included volumetry tools. The corresponding results for the left hippocampus volume are shown in Figure 8 in the manuscript.


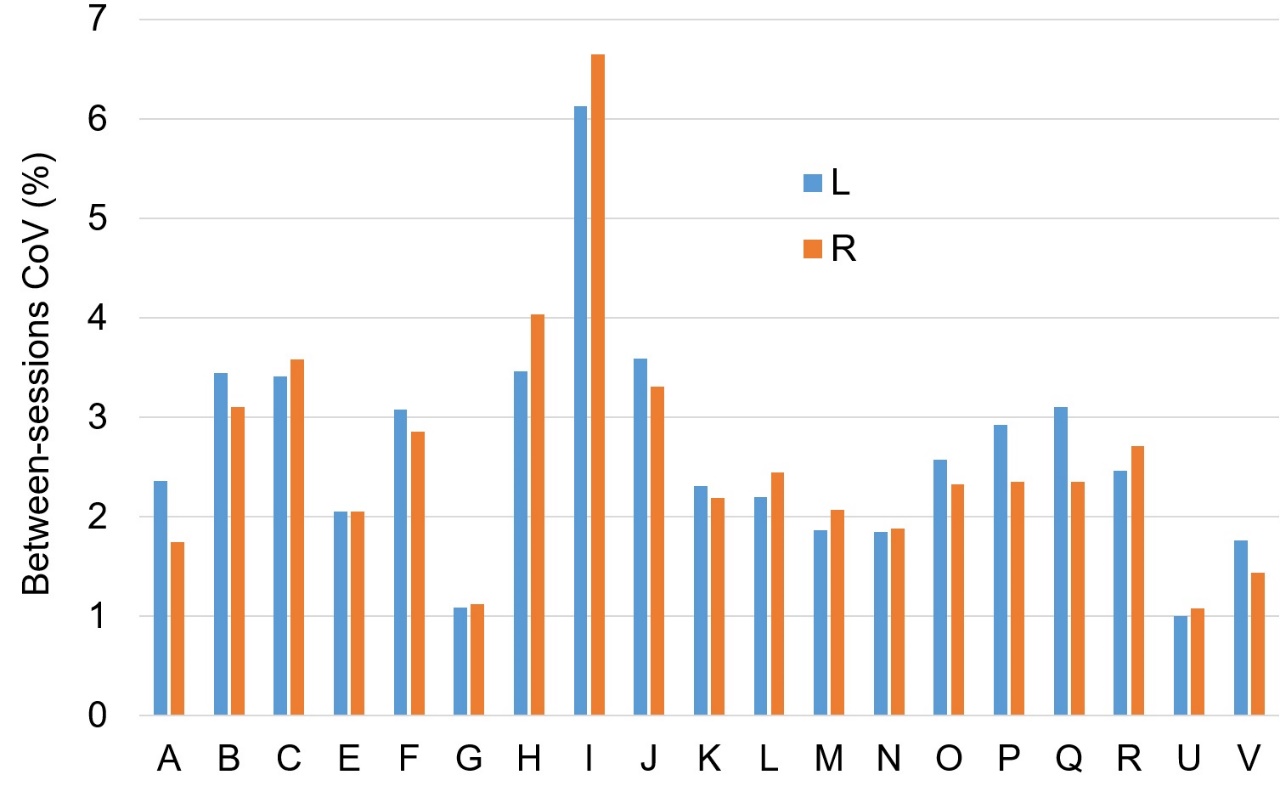


**Supplemental Figure 7.** **Sensitivity of HV estimates to the acquisition sequence.** *Between*-sessions coefficient of variation (CoV) of the within-session mean of the hippocampus volume estimate, separately for each volumetry tool (A, B,…, V) and both hemispheres (L = left, R = right). The between-sessions CoV was computed from the mean and standard deviation of the HV estimates across the non-outlier sessions shown in Supplemental Figures 3 and 4.

**Supplemental: Excluded scans**

The following scans were excluded from the analyses for the following reasons:

- secondary images from the same acquisition (derived from the primary images by post-processing): 20, 33, 45, 57, 60, 84, 123, 172, 196, 214, 235, 270, 273, 321, 366, 445, 464, 475, 485, 487, 505, 508, 526, 536, 546, 547.
- inconsistent DICOM header information (slice location attribute): 144, 220, 343.
- not acquired with a 3D sequence: 186.
- non-acceptable image quality according to visual inspection
  - very low gray-to-white matter contrast: 121, 145, 315, 447, 519.
  - severe bias field: 8, 24, 102, 128, 166, 204, 222, 236, 330, 378, 400, 514.
  - too narrow field-of-view not including the entire head: 34, 169, 185, 424, 510.
